# Phytochemical Investigation and Isolation of Chemical Constituents From the Root Extract of *Syzygium guineense* in South Ethiopia

**DOI:** 10.1002/ansa.70047

**Published:** 2025-09-25

**Authors:** Mekdes Lulu

**Affiliations:** ^1^ College of Agriculture Department of Plant Science Wachemo University Hossana Ethiopia

**Keywords:** medicinal plants, NMR spectroscopy, secondary metabolites, terpenoid, traditional medicine

## Abstract

Medicinal plants are a global healthcare cornerstone, with traditional communities, particularly in Ethiopia, relying on indigenous knowledge for treating various ailments. This study aimed to identify, isolate, and elucidate the structures of chemical constituents from the chloroform and methanol extracts of *Syzygium guineense* root using IR, 1D, and 2D NMR spectroscopic techniques in South Ethiopia of Woliata Zone. Preliminary phytochemical screening confirmed the presence of various secondary metabolites, including alkaloids, flavonoids, terpenoids, polyphenols, saponins, tannins, glycosides, and steroids. The result indicated that the powdered root material was sequentially extracted with chloroform and methanol, yielding 5 g of chloroform extract (SGA) and 9 g of methanol extract (SGB). Subsequent chromatographic fractionation of the extracts led to the isolation of two pure compounds: SGB‐3 (25 mg) from the methanol extract and SGA‐24 (100 mg) from the chloroform extract. The structural elucidation of both compounds was performed using 1D and 2D NMR spectroscopy. The spectroscopic data (IR, ^1^H NMR, ^1^ ^3^C NMR, and DEPT) of SGB‐3 indicated a diterpene lactone, characterized by hydroxyl, olefinic, and lactone functionalities. Partial characterization of SGA‐24 through 1D and 2D NMR (^1^H NMR, ^1^ ^3^C NMR, DEPT, HMBC, HSQC) revealed its identity as a terpenoid with an oleanane Δ^1^ ^2^‐ene skeleton, showing close similarities to arjunolic acid. These findings contribute valuable scientific evidence to the phytochemistry of *Syzygium guineense* roots from South Ethiopia, supporting its traditional uses and identifying potential lead compounds for further pharmacological investigations.

## 1 Introduction

Medicinal plants have served as a cornerstone of healthcare systems worldwide, providing a rich reservoir of bioactive compounds with diverse therapeutic applications. Traditional communities, particularly in regions with high biodiversity such as Ethiopia, have long relied on indigenous plant knowledge for treating various ailments [1]. This reliance is often rooted in centuries of empirical observations passed down through generations.

This continued reliance on traditional plant‐based remedies is being further validated and amplified by modern scientific inquiry. Recent advancements in analytical techniques, such as high‐performance liquid chromatography (HPLC) and nuclear magnetic resonance (NMR) spectroscopy, are enabling precise identification and characterization of bioactive compounds within these plants. Furthermore, the integration of cutting‐edge technologies like artificial intelligence (AI), machine learning (ML), and bioinformatics is revolutionizing medicinal plant research by accelerating drug discovery, predicting interactions with biological targets, and even facilitating personalized medicine based on individual genetic profiles [2, 3]. These developments are bridging the gap between traditional wisdom and contemporary pharmacology, highlighting the immense untapped potential of natural products.

In Ethiopia, specifically, recent ethnobotanical studies continue to uncover a rich diversity of medicinal plants and associated indigenous knowledge. For example, a study in the Metema district identified 85 medicinal plant species used for various ailments, with the *Fabaceae* family being particularly prominent [4]. Another investigation in the Sodo District documented 106 plant species, emphasizing the continued integral role of traditional medicine in local culture [5]. These findings not only underscore the importance of preserving this valuable biodiversity and traditional knowledge but also provide a scientific basis for further pharmacological investigations into the efficacy and safety of these traditionally used plants.

*Syzygium guineense* (Willd.) DC, a prominent species within the *Myrtaceae* family, is widely distributed across tropical and subtropical Africa, where it is locally known as “water berry” or “water‐pear” [6]. Indigenous to various parts of Africa, including Ethiopia, this plant is highly valued in traditional medicine systems for its diverse therapeutic properties. Different parts of *S. guineense*, such as the leaves, bark, fruits, and roots, have been traditionally used to treat a wide array of ailments, including gastrointestinal disorders, fevers, pain, inflammatory conditions, and various infections [7]. The extensive ethnobotanical history of *S. guineense* suggests the presence of numerous bioactive chemical constituents responsible for its observed medicinal effects.

Pharmacological investigations have increasingly supported the traditional claims of *S. guineense*. A comprehensive review published in 2025 highlights the presence of over 200 different compounds in *S. guineense*, including flavonoids, alkaloids, tannins, and terpenoids, which contribute to its diverse therapeutic properties. These studies have reported strong antibacterial, antimalarial, antihypertensive, anti‐tuberculosis, anthelmintic, anti‐venom, antiulcer, analgesic, anti‐inflammatory, and anti‐diabetic activities [2, 3]. For instance, the stem bark extract has shown promising antibacterial activity against *Staphylococcus aureus* and *Escherichia coli*, while leaf extracts have exhibited significant antioxidant and anti‐inflammatory effects, attributed to their high phenolic content (*Syzyguim guineense* Extracts Show Antioxidant Activities and Beneficial Activities on Oxidative Stress Induced by Ferric Chloride in the Liver Homogenate—PubMed Central, Phytochemical Investigation of Bioactive Components from the Stem Bark of *S. guineense* for Antibacterial and Antioxidant Conducts—ResearchGate). These scientific validations provide a strong basis for further drug development from this valuable plant.

Despite the promising preclinical results, well‐designed clinical trials are crucial to confirm the safety and efficacy of *S. guineense* in humans. While studies have indicated a generally safe profile at acute doses, sub‐chronic toxicity tests in rats have shown that high doses may affect food consumption, weight gain, and lead to increased serum levels of liver and kidney enzymes, suggesting potential toxicity with liberal, long‐term consumption. Therefore, judicious and regulated use, alongside further research, is imperative to harness the full medicinal potential of *S. guineense* while ensuring patient safety. This plant exemplifies the need for a balanced approach that combines traditional knowledge with rigorous scientific validation for the development of new, effective, and safe phytomedicines.

Previous research has extensively explored the phytochemical profiles and pharmacological activities of *S. guineense* from various geographical locations and plant parts. Studies have reported the isolation of flavonoids, tannins, triterpenes, and other phenolic compounds from the leaves and bark, demonstrating antioxidant, anti‐inflammatory, antimicrobial, and antidiabetic properties [8, 9, 10]. For instance, investigations into fruit extracts have revealed significant antioxidant capacities attributed to their high anthocyanin content [11]. While these studies have provided valuable insights into the plant's chemical composition and biological activities, there remains a notable gap in the comprehensive understanding of the specific chemical constituents present in the roots of *S. guineense* originating from South Ethiopia.

The widespread traditional use of *S. guineense* roots in local communities for various medicinal purposes [7], detailed phytochemical investigations focusing on this particular plant part from the South Ethiopian region are limited. Existing literature often generalizes findings across different *Syzygium* species or focuses on other parts of *S. guineense* from different regions [12], leaving a void regarding the unique chemical fingerprint of the roots from this specific geographical area. This lack of detailed chemical characterization presents a significant challenge. Without identifying the active compounds, it is difficult to standardize traditional remedies, ascertain their safety profiles, or develop new, evidence‐based phytomedicines. Consequently, the full therapeutic potential of *S. guineense* root remains largely untapped, highlighting an urgent need for systematic isolation and structural elucidation of its chemical constituents.

This research gap regarding *S. guineense* rooting from South Ethiopia is significant, especially considering the extensive traditional use of this specific plant part in the region for various ailments, including epilepsy and intestinal parasites [13]. While general phytochemical screenings of *S. guineense* roots from other regions have indicated the presence of steroids, terpenoids, saponins, flavonoids, tannins, alkaloids, phenols, and glycosides, on phytochemical investigation, the root extract of *S. guineense* and isolation of 2,3,23‐trihydroxy methyl oleanate [14]. A detailed analysis of roots from South Ethiopia would not only confirm the presence and concentration of these known bioactive compounds but could also lead to the discovery of novel compounds unique to this particular regional variant, potentially explaining its specific traditional applications. Therefore, targeted phytochemical studies involving advanced separation and spectroscopic techniques (LC‐MS, GC‐MS, and NMR) are imperative to elucidate the complete chemical profile of *S. guineense* roots from South Ethiopia. Such investigations would pave the way for validating the ethnobotanical claims through evidence‐based research, allowing for the isolation and characterization of lead compounds for potential drug development [15]. Furthermore, understanding the specific chemical fingerprint of these roots would facilitate the establishment of quality control standards for traditional preparations, ensuring their consistency, efficacy, and safety for the communities that rely on them. This focused approach is critical for translating traditional knowledge into modern therapeutic interventions and contributing to the global pharmacopeia.

This research holds profound significance, addressing critical gaps in the scientific understanding of *S. guineense* and contributing to both local and global knowledge in South Ethiopia of Wolita Zone. By systematically isolating and characterizing chemical compounds from the root of *S. guineense* that can either support or refute the traditional claims associated with its medicinal use in the Wolaita Zone. This validation is crucial for promoting informed healthcare practices and potentially leading to the development of novel pharmaceutical agents from natural sources. Given the limited reported phytochemical studies on the root of *S. guineense* from this specific geographical region, the findings will establish a foundational chemical profile. Therefore, the objective of this research was to identify, isolate, and elucidate the structures of chemical constituents from the chloroform and methanol extracts of *S. guineense* root using IR, 1D, and 2D NMR spectroscopic techniques in South Ethiopia of Woliata Zone.

## 2 Materials and Methods

### 2.1 Plant Material Collection and Identification

**FIGURE 1:**
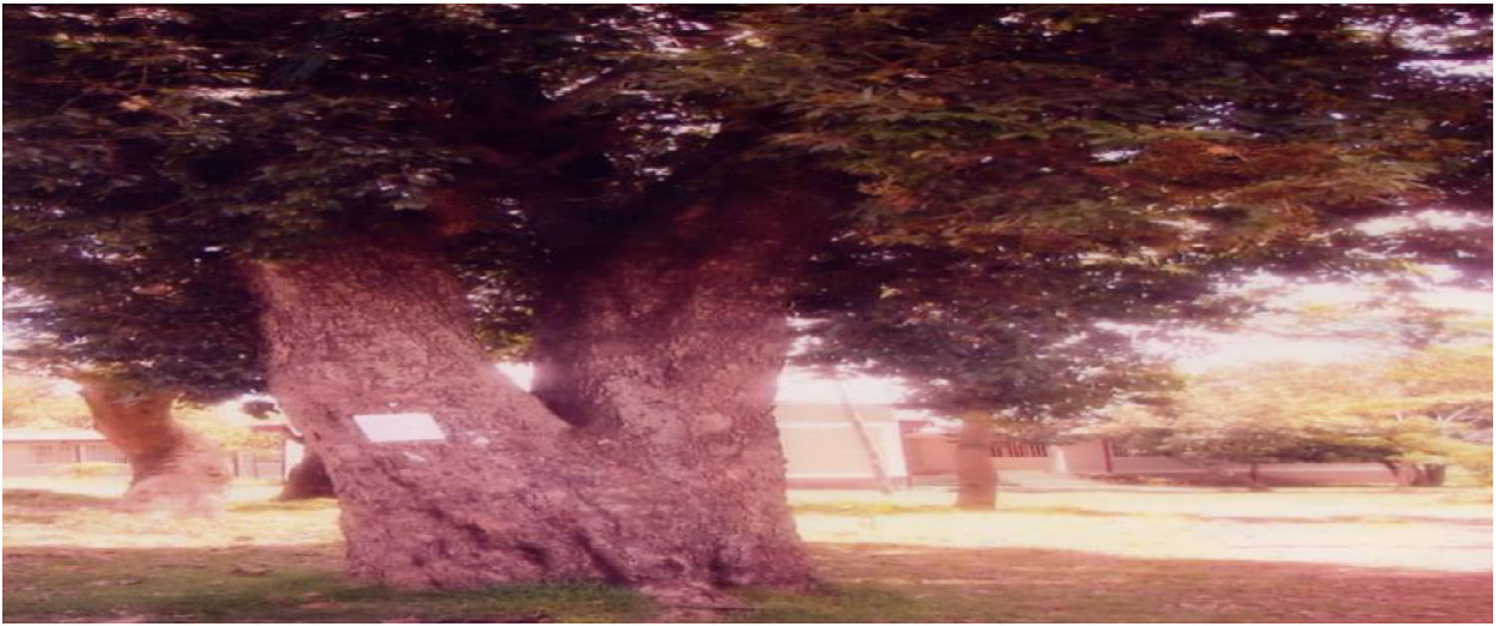
Live plant of Dokma in Woliata zone, Damot pulassa district, Zamine village.

The root of *S. guineense* was collected from the Zamine village administrative council within the Damot Pulassa district, Wolaita Zone, South Ethiopia, and is presented in Figure 1. Damot Pulassa district is located in the Wolaita Zone of the South Ethiopia Regional State. It lies approximately 388 km southwest of Addis Ababa, the capital of Ethiopia. Geographically, Damot Pulassa is situated between 6°95′–7°11′N latitude and 37°96′–38°46′E longitude, with its administrative center being Shanto. The altitude of the district ranges from 1200 to 1500 m above sea level, and it is largely categorized as “weyna‐dega” (midland agro‐ecological zone), with both dry and humid sub‐zones.

*Syzygium* is a genus of flowering plants derived from the Greek “syzygios” (“paired”), referring to the paired leaves and twigs found in many species. The species name *guineense* signifies that the tree was first collected in Guinea. It is commonly known as water berry, water pear, and water snake in English, and has local names in Ethiopia, including “*Dokma*” (Amharic), “*Ocha*” (Wolayttatto), “*Karava*” (Gurage), and “*liham*” (Tigrigna) [14, 16]. *S. guineense* is a medium‐to‐tall evergreen tree, with some specimens reaching up to 30 m in height. Its bark is often flaky and grayish‐white. The tree has broadly lanceolate leaves arranged oppositely on the branches and produces an ellipsoid, purplish drupe fruit. It typically grows in moist conditions and lowland rainforests at altitudes ranging up to 2700 meters [17].

Climate data for the Wolaita Zone and surrounding areas indicate significant rainfall variability and a warming trend. The mean annual rainfall in the district is approximately 1450 mm, though historical data show considerable fluctuation, with recorded annual rainfall varying between 703.1 and 1362.35 mm. Rainfall distribution is bimodal, with the highest precipitation typically occurring during the summer months (June, July, and August). The daily average temperature in Damot Pulassa is around 22.5°C. Over the past two decades, average temperatures in the broader Wolaita Zone have risen by about 1.3°C, and rainfall patterns have become more unpredictable, leading to both prolonged dry spells and occasional flooding.

The Wolaita Zone in South Ethiopia is rich in traditional medicinal plant knowledge, with communities relying heavily on diverse flora to treat various human and livestock ailments. Numerous ethnobotanical studies have documented this extensive use. While a comprehensive list would be vast, here are some examples of medicinal plants and their reported uses in the Wolaita Zone. The Wolaita Zone in South Ethiopia boasts a rich tradition of medicinal plant use, with communities utilizing various species for diverse ailments. Key examples include *Ajuga integrifolia* and *Artemisia afra* for stomach ache, *Allium sativum* and *Moringa stenopetala* for malaria, and aloe vera for malaria and diabetes. Traditional remedies frequently employ leaves, roots, and barks, prepared through methods like crushing and boiling, and administered orally or topically. While plant families such as *Lamiaceae* and *Asteraceae* are dominant, this invaluable ethnomedicinal knowledge faces threats from agricultural expansion and deforestation, with concerns growing over the intergenerational transfer of this vital heritage.

### 2.2 Preliminary Phytochemical Screening

Preliminary phytochemical screening of the methanol and chloroform extracts from the powdered root of *S. guineense* was conducted following standard analytical procedures described by Trease and Evans [18]. This analysis revealed the presence of alkaloids, flavonoids, terpenoids, polyphenols, saponins, tannins, glycosides, and steroid compounds. Anthraquinones, however, were notably absent. The detailed results of these tests are summarized in Table 1.

**TABLE 1:** Preliminary phytochemical screening of the powdered root of *Syzygium guineense*.

Secondary metabolite	Reagent/Procedure used	Result
Terpenoids	2‐4‐DNPH	+ve
Alkaloids	Mayer's reagent + Dragendorff's reagent	+ve
Flavonoids	1 mL of dilute ammonia solution + NaOH	+ve
Saponins	Honeycomb froth formation or Warming in water bath	+ve
Steroids	Acetic anhydride in ice + CHCl_3_ and Conc. H_2_SO_4_	+ve
Tannins	1% K_3_Fe(CN)_6_ + Conc. NH_3_	+ve
Glycosides	Fehling's solution	+ve
Phytosterols	7% H_2_SO_4_ and Benedict's solution	+ve
Anthraquinones	Benzene + 10% NH_3_ solution	−ve

*Note*: +ve and −ve signs indicate the presence and absence of the particular secondary metabolites, respectively.

#### 2.2.1 Screening Procedures

As presented in Table 1, the preliminary phytochemical screening involved specific tests to identify various secondary metabolites. Alkaloids were detected by adding HCl and Dragendorff's reagent to the extract, with an orange or red precipitate confirming their presence. Flavonoids were identified by an intense yellow color upon adding dilute sodium hydroxide, which became colorless after acidification. Glycosides were indicated by a pink to red color after acid hydrolysis, followed by the addition of pyridine, sodium nitroprusside, and sodium hydroxide. Phytosterols produced a bluish‐green color after refluxing the extract with alcoholic potassium hydroxide, extracting with ether, and then adding diluted acetic acid, acetic anhydride, and concentrated H_2_SO_4_. Saponins were confirmed by the formation of a 1 cm layer of foam upon diluting the extract with distilled water and agitating. For steroids, a red upper layer and a yellow layer with a green fluorescent sulfuric acid layer indicated their presence after dissolving the extract in chloroform and carefully adding concentrated sulfuric acid. Tannins were detected by a yellow precipitate formed when the extract was mixed with 1% lead acetate solution. Triterpenoids yielded a reddish‐violet color in the Salkowski test, which involved chloroform, acetic anhydride, and concentrated H_2_SO_4_. Finally, anthraquinones were tested by shaking the crude powder with benzene and adding ammonia solution, with the absence of a violet color confirming their absence.

### 2.3 Coding System

A specific coding system was employed for the isolated compounds to ensure clear identification and traceability: ‘S’ denotes the genus *Syzygium*, and ‘G’ denotes the species *guineense*. ‘A’ and ‘B’ represent the chloroform and methanol extracts, respectively, from which the compounds were isolated. The numerical suffix following SG‐A or SG‐B indicates the unique pure compound isolated from that specific extract, allowing for precise referencing of each isolated constituent throughout the study.

### 2.4 Experimental Procedures

Infrared (IR) spectra were recorded as KBr pellets on a Perkin–Elmer BX IR spectrometer, spanning the range of 4000–400 cm^−1^. One‐dimensional (^1^H NMR, ^1^ ^3^C NMR) and two‐dimensional nuclear magnetic resonance (2D NMR) spectra were obtained using a Bruker Advance 400 spectrometer at 400 MHz. The purity of isolated compounds was monitored via thin‐layer chromatography (TLC) on silica gel GF254 plates (0.2 mm thick layer of Merck silica gel). For compound fractionation, column chromatography was performed using silica gel 60 (Merck) with a particle size of 0.063–0.200 mm (70–230 mesh ASTM).

### 2.5 Preparation and Extraction of Plant Material

**SCHEME 1:**
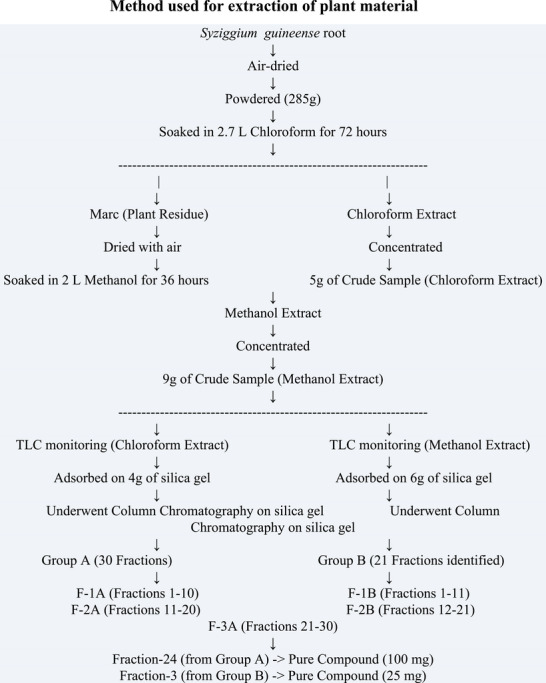
Method used for extraction of plant material.

The preparation and extraction of plant material are presented in Scheme 1. The collected root of *S. guineense* was air‐dried, cut, and chopped into small pieces. A 285 g portion of the powdered root sample was initially soaked in 2.7 L of chloroform for 72 h. After filtration, the chloroform extract was concentrated under reduced pressure using a rotary evaporator, yielding 5 g of a brown solid residue, labeled as SGA. The solvent‐free marc (residual plant material) was then air‐dried and subsequently soaked in 2 L of methanol for 36 h. Following filtration, the methanol extract was concentrated under reduced pressure, affording 9 g of a red solid residue, labeled as SGB.

### 2.6 Isolation

#### 2.6.1 Fractionation of Chloroform Extract (Group A)

Five grams of the crude SGA extract were adsorbed onto 4.0 g of silica gel by dissolving the sample in chloroform and then drying the solvent. The dry, adsorbed sample was applied to a column packed with 80 g of silica gel, pre‐equilibrated with petroleum ether. Isolation was performed by eluting the column with solvents of increasing polarity: n‐hexane, chloroform, and ethyl acetate. A total of 30 fractions, each with a volume of 30 mL, were collected (Table 2).

**TABLE 2:** Solvent systems used in column chromatography for fractionation (Group A).

Fractions	Solvent system	Ratio	Volume	Remark
1	n‐Hexane	100%	30 mL	SGA‐1
2	n‐Hexane	»	»	SGA‐2
3	n‐Hexane	»	»	SGA‐3
4	n‐Hexane	»	»	SGA‐4
5	n‐Hexane	»	»	SGA‐5
6	n‐Hexane	»	»	SGA‐6
7	n‐Hexane	»	»	SGA‐7
8	n‐Hexane	»	»	SGA‐8
9	n‐Hexane	»	»	SGA‐9
10	n‐Hexane	»	»	SGA‐10
11	n‐Hexane: CHCl_3_	27: 3	»	SGA‐11
12	n‐Hexane: CHCl_3_	24: 6	»	SGA‐12
13	n‐Hexane: CHCl_3_	21: 9	»	SGA‐13
14	n‐Hexane: CHCl_3_	18: 12	»	SGA‐14
15	n‐Hexane: CHCl_3_	15: 15	»	SGA‐15
16	n‐Hexane: CHCl_3_	12: 18	»	SGA‐16
17	n‐Hexane: CHCl_3_	9: 21	»	SGA‐17
18	n‐Hexane: CHCl_3_	6: 24	»	SGA‐18
19	n‐Hexane: CHCl_3_	3: 27	»	SGA‐19
20	CHCl_3_	100%	»	SGA‐20
21	CHCl_3_: EtOAC	27: 3	»	SGA‐21
22	CHCl_3_:EtOAC	24: 6	»	SGA‐22
23	CHCl_3_:EtOAC	21: 9	»	SGA‐23
24	CHCl_3_:EtOAC	18: 12	»	SGA‐24,Pure
25	CHCl_3_:EtOAC	15: 15	»	SGA‐25
26	CHCl_3_:EtOAC	12: 18	»	SGA‐26
27	CHCl_3_:EtOAC	9: 21	»	SGA‐27
28	CHCl_3_:EtOAC	6: 24	»	SGA‐28
29	CHCl_3_:EtOAC	3: 27	»	SGA‐29
30	EtOAC	100%	»	SGA‐30

The elution sequence was as follows: Fractions 1–10 (Fr‐1A) were eluted with 100% n‐hexane. Fractions 11–20 (Fr‐2A) were eluted with n‐Hexane:CHCl_3_ mixtures with increasing chloroform concentration (from 27:3 to 3:27). Fractions 21–30 (Fr‐3A) were eluted with CHCl_3_:EtOAc mixtures with increasing ethyl acetate concentration.

Upon TLC analysis, fractions 1‐14 were discarded due to the absence of spots. Fractions 15‐23, which showed similar *R*_f_ values and characteristic colors on TLC, were combined, but still did not yield a single, separate spot. Fraction 24, however, showed a single pure spot when analyzed by TLC using CHCl_3_ EtOAc (30:70) as the solvent system. This fraction (Fraction 24) was left in a hood for 24 h to concentrate, yielding 100 mg of a colorless solid sample, which was labeled as compound SGA‐24. Compound SGA‐24 was then subjected to spectral analysis. The remaining fractions 25–30 showed more than two spots.

#### 2.6.2 Fractionation of Methanol Extract (Group B)

Nine grams of the crude SGB methanol extract were adsorbed onto 6.0 g of silica gel by dissolving the sample in chloroform and drying the solvent. The adsorbed sample was then applied to a column packed with 80 g of silica gel, pre‐equilibrated with petroleum ether. Successive elution with solvents of increasing polarity was performed, yielding a total of 21 fractions (Table 3).

**TABLE 3:** Solvent systems used in column chromatography for fractionation (Group B).

Fractions	Solvent system	Ratio	Volume	Remark
1	CHCl_3_	100%	30 mL	SGB‐1
2	CHCl_3_:EtOAC	27:3	»	SGB‐2
3	CHCl_3_:EtOAC	24:6	»	SGB‐3, pure
4	CHCl_3_:EtOAC	21:9	»	SGB‐4
5	CHCl_3_:EtOAC	18:12	»	SGB‐5
6	CHCl_3_:EtOAC	15:15	»	SGB‐6
7	CHCl_3_:EtOAC	12:18	»	SGB‐7
8	CHCl_3_:EtOAC	9:21	»	SGB‐8
9	CHCl_3_:EtOAC	6:24	»	SGB‐9
10	CHCl_3_:EtOAC	3:27	»	SGB‐10
11	EtOAC	100%	»	SGB‐11
12	EtOAC: EtOH	27:3	»	SGB‐12
13	EtOAC: EtOH	24:6	»	SGB‐13
14	EtOAC: EtOH	21:9	»	SGB‐14
15	EtOAC: EtOH	18:12	»	SGB‐15
16	EtOAC: EtOH	15:15	»	SGB‐16
17	EtOAC: EtOH	12:18	»	SGB‐17
18	EtOAC: EtOH	9: 21	»	SGB‐18
19	EtOAC: EtOH	6: 24	»	SGB‐19
20	EtOAC: EtOH	3: 27	»	SGB‐20
21	EtOH	100%	»	SGB‐21

The column was eluted with the following solvent systems: Fractions 1‐11 (Fr‐1B) were eluted with CHCl_3_:EtOAc mixtures with increasing ethyl acetate concentration. Fractions 12‐21 (Fr‐2B) were eluted with EtOAc:EtOH mixtures with increasing ethanol concentration.

Upon TLC analysis, all fractions except Fraction 3 were discarded as they did not show any distinct spots. Fraction 3, when processed with a CHCl_3_:EtOAc (20:80) solvent system for TLC, showed a single pure spot. This fraction was left in a hood for 48 h, during which colorless solids were observed. This solid sample, weighing 25 mg, was labeled as compound SGB‐3 and subsequently sent for spectral analysis.

### 2.7 Isolation

#### 2.7.1 Fractionation of Chloroform Extract (Group A)

Around 5 g sample of the SGA was adsorbed on 4.0 g of silica gel by dissolving the sample in chloroform. After drying the solvent, the dry sample was applied to a column packed with 80 g of silica gel with petroleum ether. Isolation was carried out using the solvents hexane, chloroform, and ethyl acetate by increasing polarity. A total of 30 fractions were obtained, each with a total volume of 30 mL (Table 2). The column was eluted with the following solvent system fractions: Fr‐1A (Fraction 1–10) with pure n‐hexane, followed by Fr‐2A (Fraction 11–20) with n‐hexane: CHCl_3_, and finally Fr‐3A (fraction 21–30) with CHCl_3_: EtOAC. Out of 30 fractions (Group A, Table 2), fractions 1–14 were discarded because their TLC result did not show spots, fractions 15–23 that showed the same *R*_f_ value and the same characteristic color on TLC were combined but still they did not show any separate single spot, fraction 24 showed pure single spot and for the remaining fractions 25–30, it was observed more than 2 spots.

Among Group A, Fraction 24 also showed a pure spot was seen on TLC using the solvent system CHCl_3_: EtOAC (30:70). The Fraction 24 was left in a hood for 24 h to concentrate the fraction, and after 24 h, the fraction gave 100 mg of the colorless solid sample, and this solid sample was labeled as compound SGA‐24. The compound SGA‐24 was sent for spectral analysis.

#### 2.7.2 Fractionation of Methanol Extract (Group B)

The crude methanol extract, 9 g of the SGB, was adsorbed on 6.0 g of silica gel by dissolving the sample with chloroform. After drying the solvent, the adsorbed sample was applied to column chromatography in which 80 g of silica gel was packed with petroleum ether. Successive elution with increasing polarity was done, and 21 fractions were obtained (Table 3). The column was eluted with the following solvent system fractions: Fr‐1B (Fraction 1–11) with CHCl_3_:EtOAC, followed by Fr‐2B (Fraction 12–21) with EtOAC: EtOH. The fractions collected, other than 3, were discarded because their TLC results did not show any spots. Fraction 3, which was processed with CHCl_3_:EtOAC (20:80) solvent system for TLC, has shown a single spot. This fraction was left in the hood for 48 h, while colorless solids were observed and then labeled as the SGB‐3 compound. The amount obtained was 25 mg, and this compound was sent for spectral analysis.

## 3 Results and Discussion

### 3.1 Solvent Extraction and Isolation of Chemical Constituents From *Syzygium guineense* Root

From the chloroform and methanol extracts of *S. guineense* root, two pure compounds, designated SGA‐24 and SGB‐3, were successfully isolated and characterized. The structural elucidation of these compounds was achieved through a meticulous analysis of their spectroscopic data. This involved a combination of one‐dimensional NMR techniques (^1^H NMR for proton environments, ^1^ ^3^C NMR for carbon skeletons, and DEPT for carbon multiplicity), and advanced two‐dimensional NMR experiments (HMBC for long‐range carbon–proton correlations and HSQC for direct carbon–proton correlations), alongside IR spectroscopy to identify characteristic functional groups. The derived spectroscopic data were then rigorously compared with published literature for known compounds, enabling the confirmation of their proposed chemical structures.

#### 3.1.1 Characterization of Compound SGB‐3 Identified It as a Diterpene Lactone

Compound SGB‐3, isolated from the methanol (CH_3_OH) extract, was obtained as a colorless powdery solid. It exhibited an *R*_f_ value of 0.54 when analyzed by thin layer chromatography (TLC) using a chloroform:ethyl acetate (20:80) solvent system, appearing as a sharp, well‐defined single spot. The compound was readily soluble in chloroform, aligning with common characteristics of isolated secondary metabolites from *Syzygium* species [19].

**FIGURE 2:**
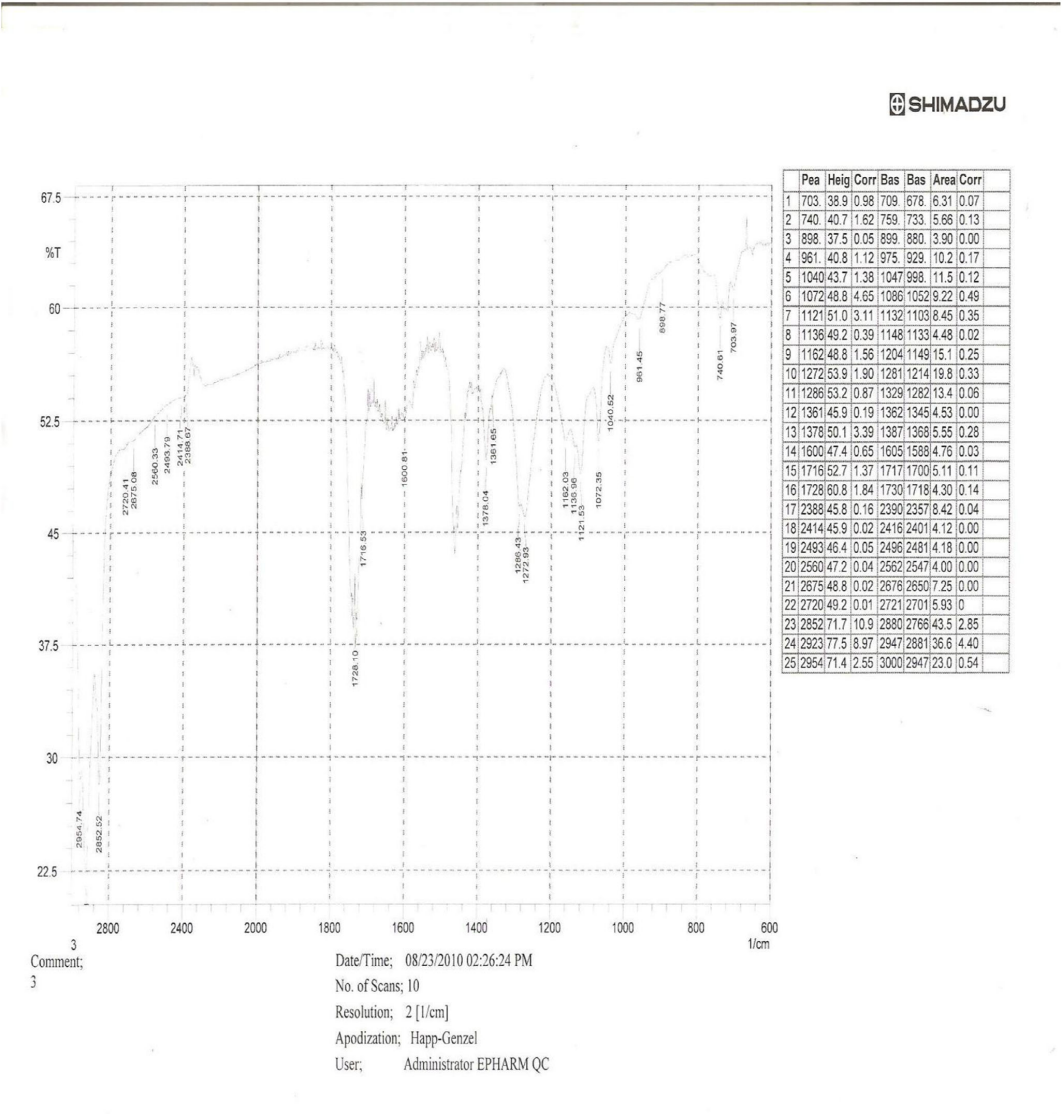
IR spectrum of SGB‐3.

**IR Spectrum Analysis** (Infrared Spectrum Analysis): The IR (KBr) spectrum (Figure 2) of SGB‐3 displayed a broad absorption band at 3400 cm^−1^, characteristic of a hydroxyl group. An obscured absorption band at 3010 cm^−1^ suggested the presence of olefinic C─H bond stretching. Strong absorption at 2900 cm^−1^ and medium absorptions at 2850.8, 2852.5, and 2853 cm^−1^ indicated the presence of saturated C─H groups. Two weak absorption bands at 1600 and 1570 cm^−1^ were consistent with alkene functionalities. A strong absorption band at 1740 cm^−1^ indicated the presence of a lactone group. The presence of these functional groups is consistent with the general characteristics of diterpene lactones, which often feature hydroxyl groups, olefinic bonds, and cyclic ester (lactone) moieties, as supported by recent spectroscopic studies on similar compounds [20, 21].

**FIGURE 3:**
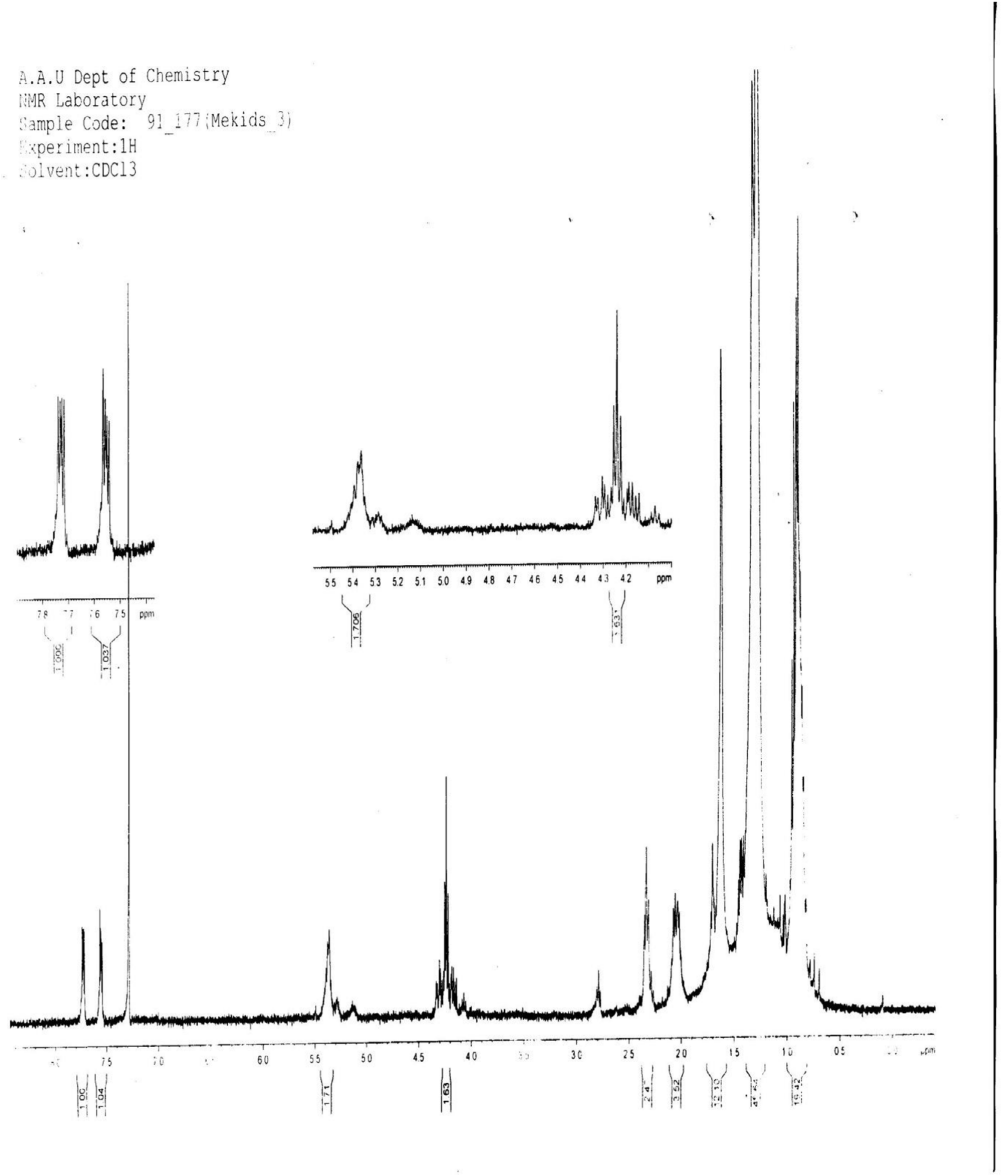
1H spectrum of SGB‐3.

**^1^H NMR Spectrum Analysis** (Proton Nuclear Magnetic Resonance Spectrum Analysis): The ^1^H NMR spectrum (Figure 3, Table 4) of SGB‐3 in CDCl_3_ provided crucial insights into its structure. The proton (1H) NMR, a standard format, is used to present chemical shift, multiplicity, and integration. A complete entry should include the chemical shift (*δ* in ppm), followed by the multiplicity (s, d, t, q, m) and the integration (number of protons) in parentheses. The entry for C13 is correctly written as 1.6 (d, 1H), meaning a signal at 1.6 ppm that is a doublet and corresponds to one proton. Similarly, C17 is 4.23 (m, 2H), indicating a signal at 4.23 ppm that is a multiplet and corresponds to two protons. The entries for carbons 4, 5, 6, 9, 10, 18, 21, 22, and 23 are incomplete because they are missing the integration value, which is critical for determining the number of protons for each signal.

**TABLE 4:** ^1^H, ^1^ ^3^C NMR and DEPT Spectra Data of Compound SGB‐3 in CDCl_3._

Carbon and Hydrogen Number	^1^H *δ* (ppm)	^1^ ^3^C *δ* (ppm)	^1^ ^3^C/DEPT
1	—	168.1	Quaternary carbon
2	—	132.6	Quaternary carbon
3	—	130.1	Quaternary carbon
4	1.1–1.5 (m, **2H**)	32.0	CH_2_
5	1.1–1.5 (m, **2H**)	30.4	CH_2_
6	1.1–1.5 (m, **2H**)	29.7	CH_2_
7	—	34.1	Quaternary carbon
8	—	29.6	Quaternary carbon
9	1.1–1.5 (m, **2H**)	29.4	CH_2_
10	1.1–1.5 (m, **2H**)	29.1	CH_2_
11	—	129.7	Quaternary carbon
12	—	128.1	Quaternary carbon
13	1.6 (d, 1H)	68.1	CH
14	7.75 (dd, 1H)	130.9	CH
15	7.56 (dd, 1H)	128.9	CH
16	5.35 (dt, 1H)	38.0	CH
17	4.23 (m, 2H)	28.9	CH_2_
18	1.0–1.5 (m, **2H**)	23.7	CH_2_
19	2.35 (quintet, 2H)	23.0	CH_2_
20	2.0 (sextet, 2H)	22.7	CH_2_
21	0.71–0.98 (m, **3H**)	11.0	CH_3_
22	0.71–0.98 (m, **3H**)	14.2	CH_3_
23	0.71–0.98 (m, **3H**)	14.1	CH_3_

Multiplet peaks observed in the regions of *δ* 0.69–0.98 ppm and 1.0–1.5 ppm are characteristic of aliphatic methyl and methylene protons. A multiplet peak at *δ* 4.23 ppm was assigned to methylene signals, likely adjacent to an electronegative atom. A doublet peak at *δ* 1.6 ppm indicated a methine proton attached to a hydroxyl group. The sharp singlet peak at *δ* 7.25 ppm corresponded to the residual solvent (CDCl_3_). Olefinic protons appeared as a doublet of doublets at *δ* 7.61 and δ 7.76 ppm, indicative of specific unsaturation patterns. A doublet of triplets at *δ* 5.45 ppm was assigned to a methine proton that is geminal to a methylene proton and an olefinic proton, further supporting the presence of complex unsaturation and oxygenation. These chemical shifts and coupling patterns are typical for diterpenoid structures, particularly those with unsaturation and oxygenation, aligning with recent spectroscopic analyses of similar natural products [22, 23].

**FIGURE 4:**
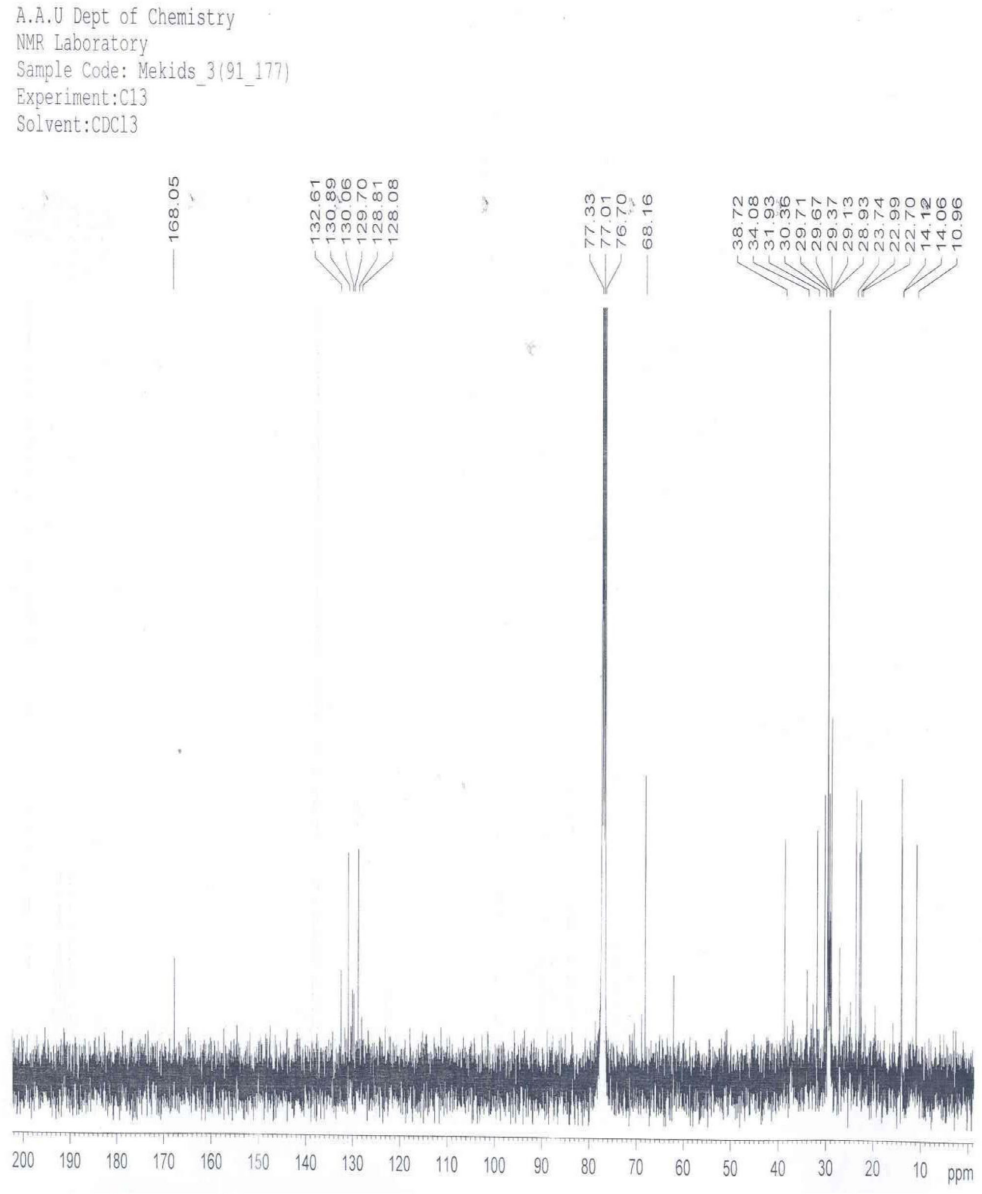
C‐13 spectrum of SGB‐3.

**^1^ ^3^C NMR Spectrum Analysis** (Carbon‐13 Nuclear Magnetic Resonance Spectrum Analysis): The ^1^ ^3^C NMR spectrum of compound SGB‐3 (Figure 4, Table 4) provided key structural information through its distinct chemical shift regions. Aliphatic carbons resonated between *δ* 11 and *δ* 68 ppm, with specific peaks observed at *δ* 11, 14.1, 14.2, 22.7, 23.0, 23.7, 28.9, 28.1, 29.1, 29.4, 29.7, 29.7, 30.4, 32, and 34.1 ppm. The solvent signal (CDCl_3_) was observed between *δ* 76.7 and *δ* 77.3 ppm. Olefinic carbons appeared in the region of *δ* 128.1 and *δ* 132 ppm, specifically at *δ* 132.6, 130.9, 130.1, 129.7, 128.1, and 128.9 ppm, confirming the presence of carbon–carbon double bonds. A characteristic peak at *δ* 168.1 ppm confirmed the presence of a lactone carbonyl carbon. The observation of 23 carbon signals, including these characteristic olefinic and carbonyl carbons, strongly supports a diterpenoid framework, which typically contains 20 carbon atoms, with additional carbons potentially arising from side chains or modifications, consistent with general principles of ^1^ ^3^C NMR spectroscopy as detailed by Farmer et al. [24].

**FIGURE 5:**
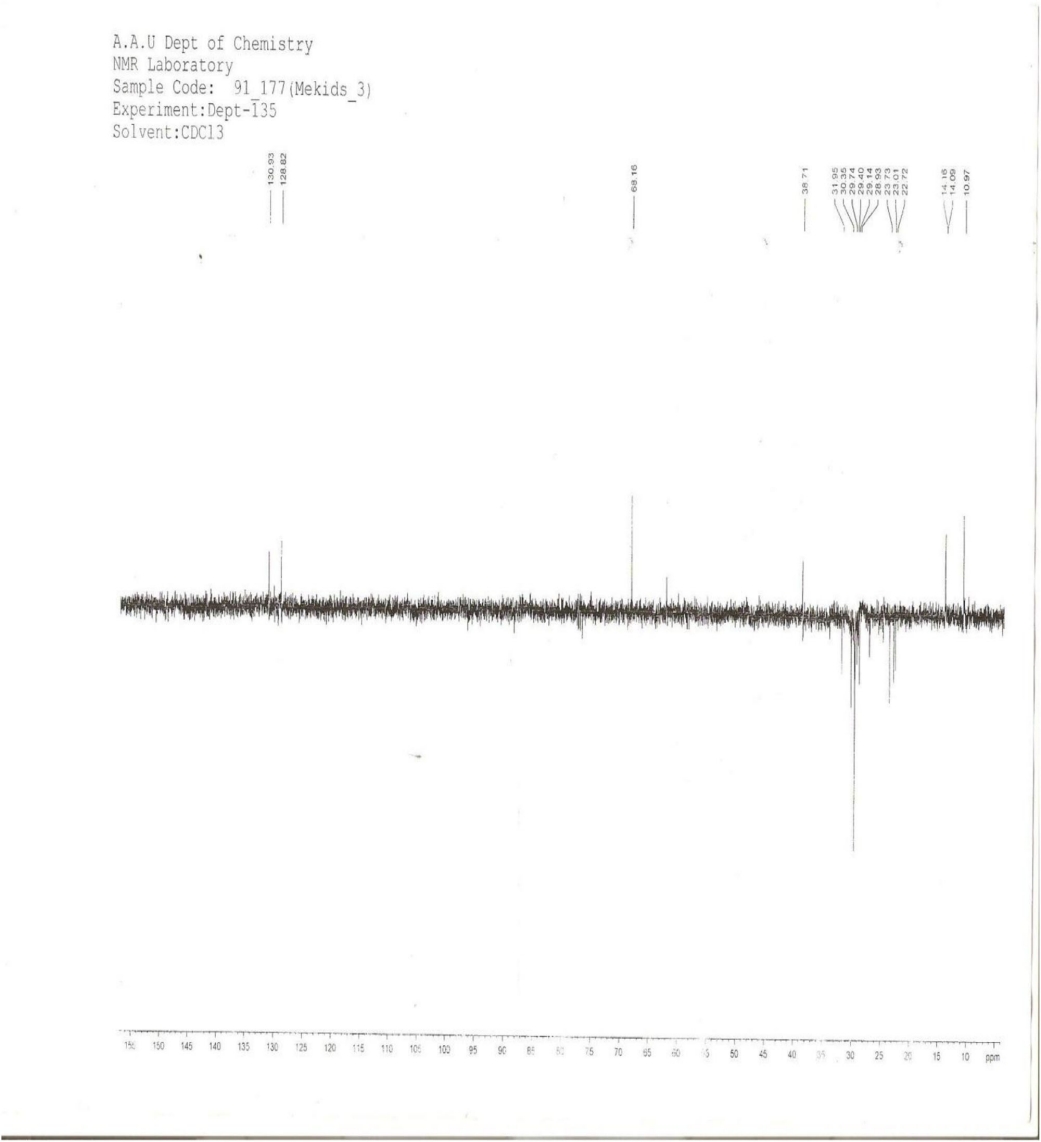
DEPT spectrum of SGB‐3.

**DEPT Spectrum Analysis** (Distortionless Enhancement by Polarization Transfer): Spectrum analysis (Figure 5, Table 4) of compound SGB‐3 provided detailed insights into the types of carbon atoms present. Out of the 23 carbons observed in the ^1^ ^3^C NMR spectrum, the DEPT analysis indicated the presence of 16 protonated carbon atoms. Specifically, it confirmed the presence of three aliphatic methyl (CH_3_) groups at *δ* 11, 14.1, and 14.2 ppm. Nine aliphatic methylene (CH_2_) groups were identified at *δ* 22.7, 23.0, 23.7, 28.9, 29.1, 29.4, 29.7, 30.4, and 32 ppm. Two aliphatic methine (CH) groups were observed at *δ* 68.2 and 38.1 ppm, with the methine carbon at *δ* 68.2 ppm being assigned as attached to a hydroxyl group, consistent with the deshielding effect of oxygen. In addition, two olefinic methine carbons were present at *δ* 128.8 and 130.9 ppm, confirming unsaturation. The difference of seven carbon atoms between the total carbons from the ^1^ ^3^C NMR and the protonated carbons from the DEPT spectra confirmed the presence of seven quaternary (C) carbon atoms at *δ* 168.1, 132.6, 130.1, 129.7, 128.1, 34.1, and 29.6 ppm. The DEPT data further confirms the nature of these carbon atoms (methyl, methylene, methine, and quaternary), which is crucial for building the molecular skeleton of a complex natural product like a diterpene lactone [25, 26, 27].

**FIGURE 6:**
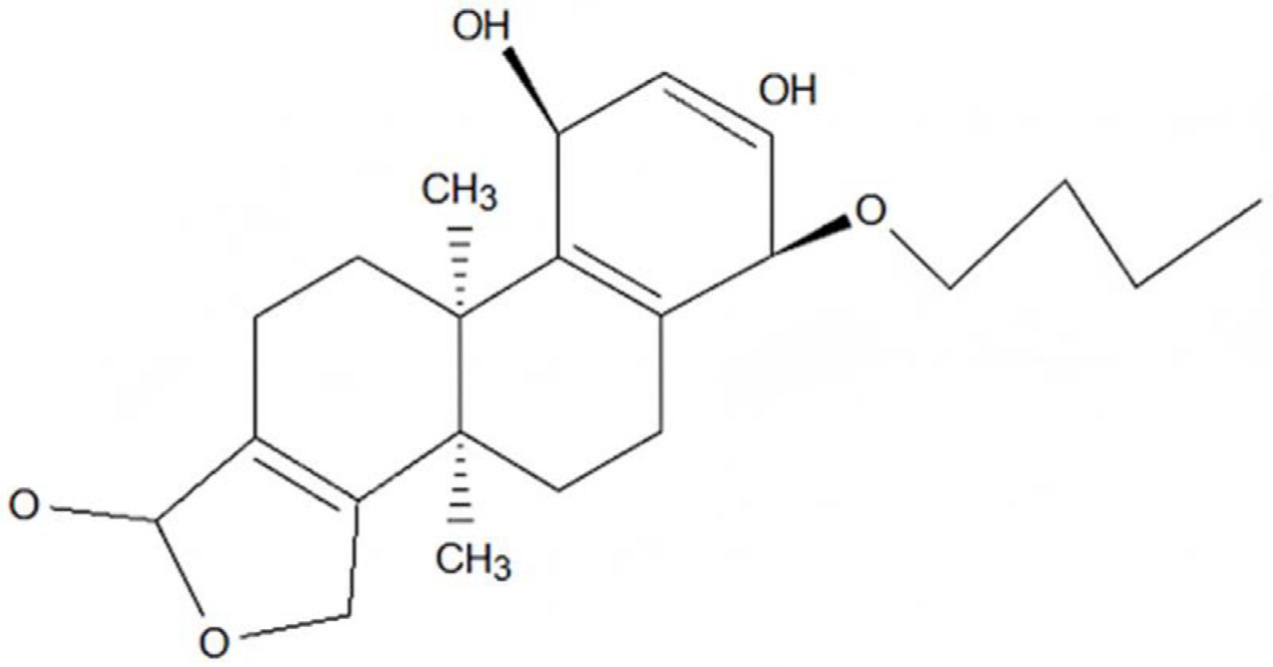
Diterpene lactone.

Based on the spectrum data from the ^1^H, ^1^ ^3^C, and DEPT NMR spectra, the proposed structure for the isolated natural product is the Diterpene Lactone, Neoandrographolide (Figure 6). The number of carbon atoms, the type of carbons (CH_3_, CH_2_, CH, and quaternary), and the specific chemical shifts of the functional groups, such as the lactone carbonyl and double bonds, all perfectly match the known properties of this compound. Recent studies continue to explore the chemical constituents and biological activities of *S. guineense*; its rich phytochemicals include terpenoids [28, 29]. Diterpene lactones are a significant class of natural products known for diverse biological activities and have been isolated from various plant sources, including the *Myrtaceae* family to which *S. guineense* belongs [30].

#### 3.1.2 Partial Characterization of Compound SGA‐24 Identified It as a Terpenoid, Showing Arjunolic Acid

Compound SGA‐24 was obtained as a colorless solid from the chloroform extract. Its partial characterization was performed using various spectroscopic techniques. This compound exhibited an *R*_f_ value of 0.68 when analyzed by TLC using a CHCl_3_:EtOAc (70:30) solvent system.

**FIGURE 7:**
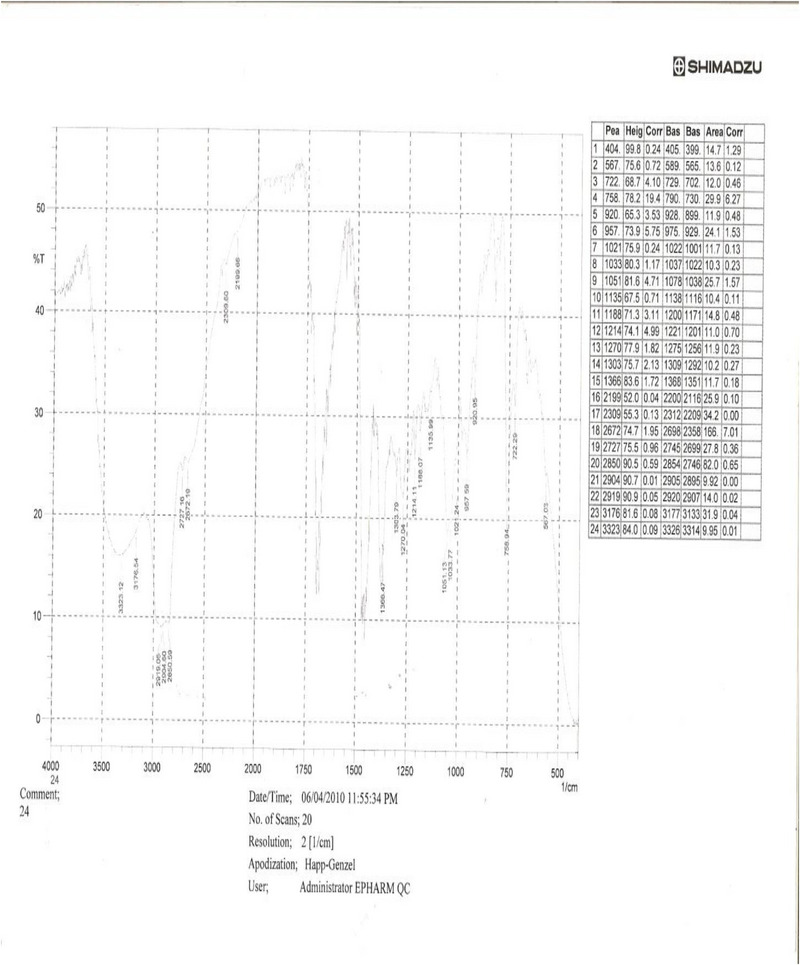
IR spectrum of SGB‐24.

**IR Spectrum Analysis**: The IR (KBr) spectrum (Figure 7) of SGA‐24 provided crucial information about its functional groups. A broad absorption band at 3440 cm^−1^ is characteristic of a hydroxyl (─OH) group, often broadened due to hydrogen bonding. A strong absorption band at 2919 cm^−1^ suggested the presence of a carboxylic acid (─COOH) functionality, which typically shows a broad O─H stretch in the 3300–2500 cm^−1^ range overlapping with C─H stretches [31]. Strong absorption bands at 2904 and 2850 cm^−1^ were indicative of saturated C─H stretching vibrations, common in alkyl chains (Liu 2023). Furthermore, a vibration frequency band at 1735 cm^−1^ indicated the presence of an olefinic group, specifically a C═O stretch that can be found in esters or lactones, which can be part of an olefinic system. These characteristic IR absorption patterns are consistent with those reported in various studies on organic compounds and natural products, including research conducted in Ethiopia on plant extracts.

**FIGURE 8:**
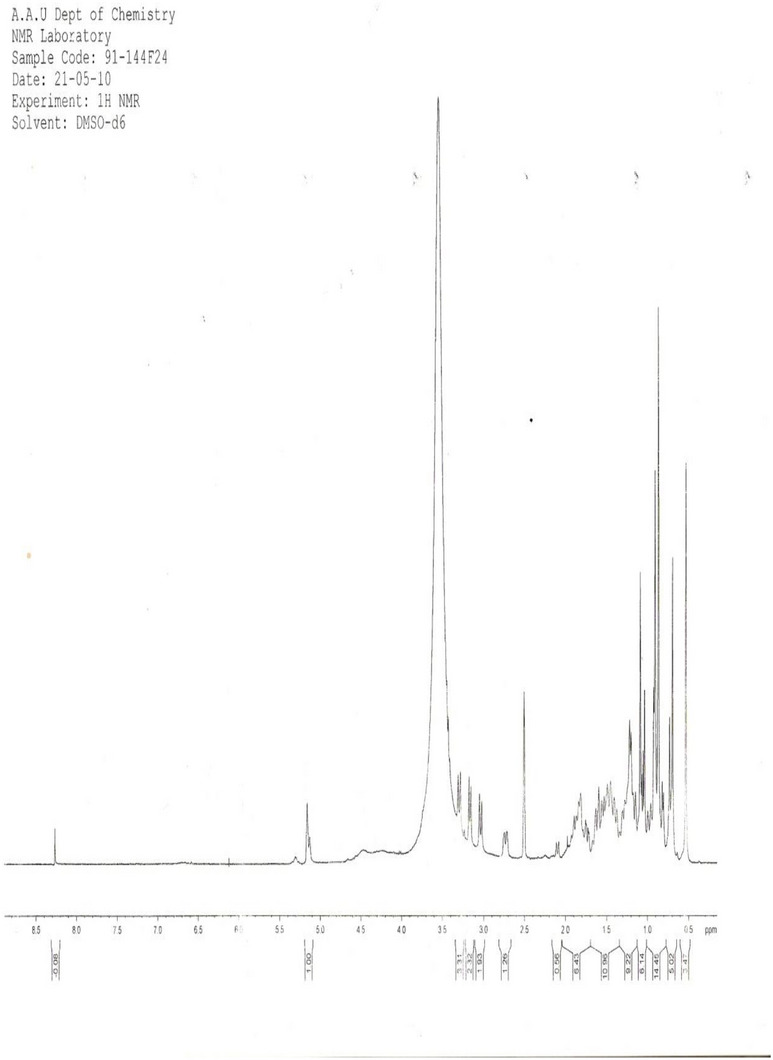
1H spectrum of SGA‐24.

**^1^H NMR Spectrum Analysis**: The ^1^H NMR spectrum (Figure 8, Table 6) of partially characterized SGA‐24 in DMSO‐d_6_ revealed key structural features. A triplet peak at *δ* 5.12 ppm was assigned to an olefinic proton, consistent with typical chemical shifts for protons on carbon–carbon double bonds [32]. A broad singlet peak at *δ* 3.5 ppm and doublet peaks at *δ* 3.29 and 3.06 ppm indicated hydroxyl‐bearing methine and methylene protons, respectively, with the broadness often attributed to hydrogen bonding. The sharp singlet peak at *δ* 2.5 ppm corresponded to the solvent (DMSO‐d_6_), a common internal standard [33]. A doublet peak at *δ* 2.74 ppm was assigned to a methine proton, falling within the expected range for such protons. Multiplet peaks between *δ* 1.3 and 1.8 ppm indicated other methine and methylene protons within the aliphatic region of the molecule. Finally, six singlet peaks for methyl groups were observed at *δ* 0.54 (3H, s), 0.86 (3H, s), 0.87 (3H, s), 0.88 (3H, s), 0.89 (3H, s), and 1.16 (3H, s) ppm, which are characteristic of the numerous methyl groups often found in complex natural products like triterpenes [34, 35]

**FIGURE 9:**
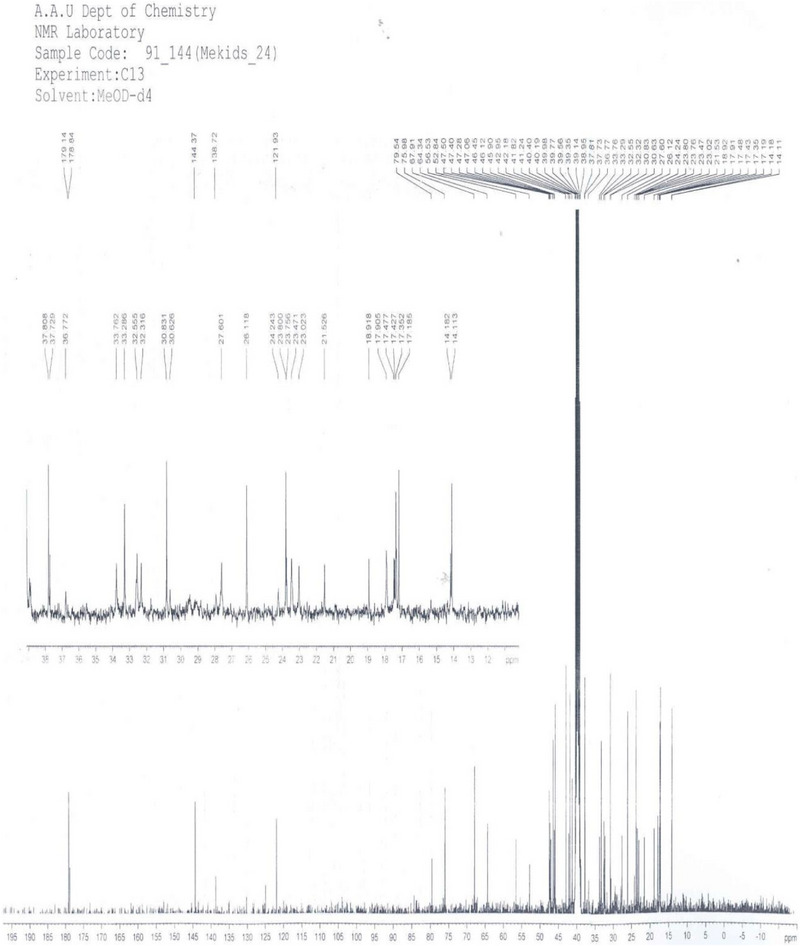
C‐13 spectrum of SGA‐24.

**FIGURE 10:**
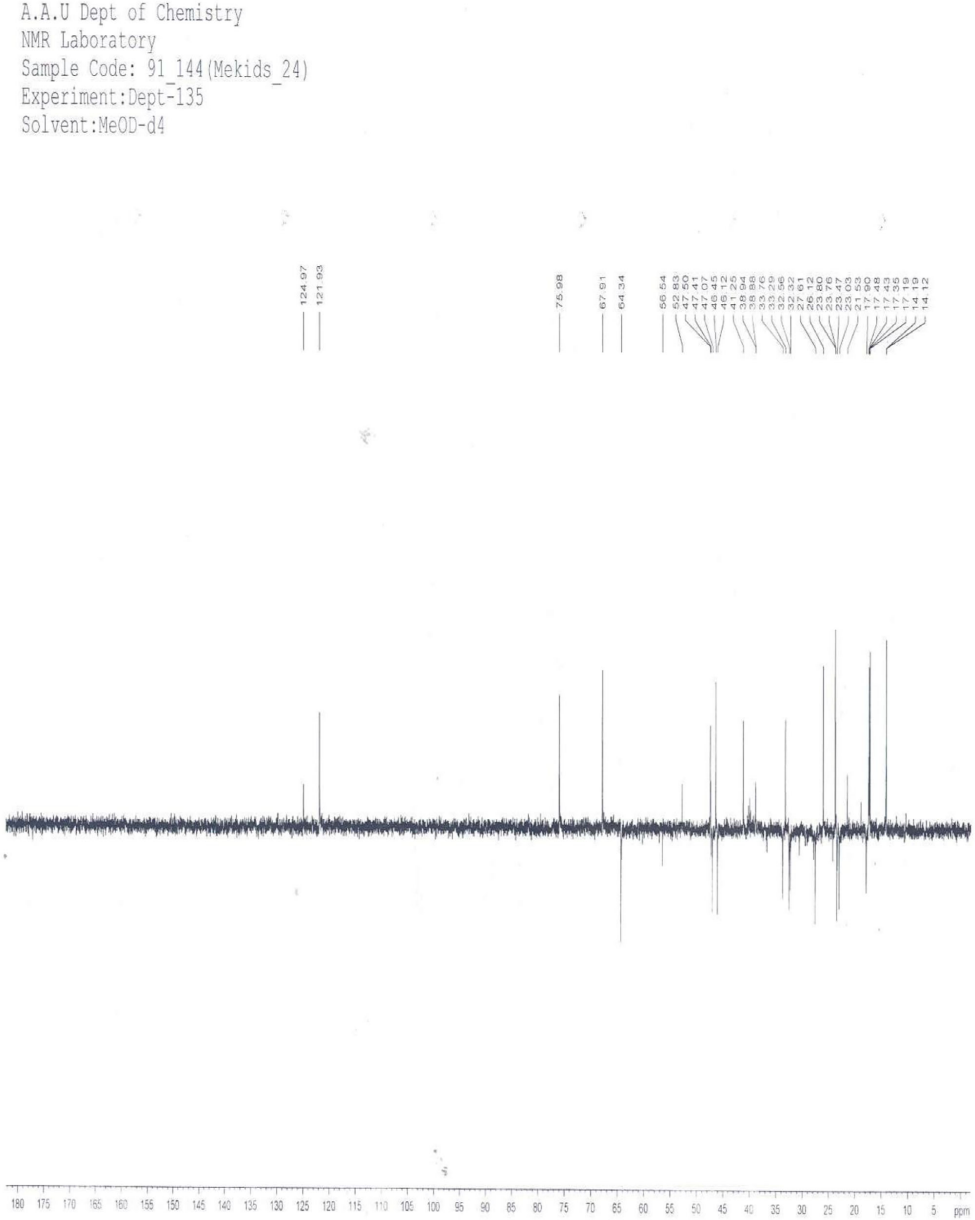
DEPT spectrum of SGA‐24.

**^1^ ^3^C NMR and DEPT‐135 Spectrum Analysis**: The ^1^ ^3^C NMR and DEPT‐135 spectra (Figures 9 and 10, Table 5) of compound SGA‐24 in DMSO‐d_6_ showed well‐resolved resonances for 47 carbon atoms. While these were compared with literature data for arjunolic acid [36], a significant number of carbons (17, from C‐31 to C‐47) remained unassigned to the partial structure of SGA‐24. This is a common challenge in the NMR analysis of complex natural products like triterpenes, where overlapping signals, low natural abundance of ^1^ ^3^C, and the presence of multiple similar environments can make complete assignment difficult, often requiring advanced 2D NMR techniques and computational methods for full elucidation [37, 38, 39].

**TABLE 5:** Comparison of ^1^ ^3^C NMR spectral data of compound SGA‐24 with arjunolic acid from literature.

Carbon No.	Literature ^1^ ^3^C *δ* (ppm)	^1^ ^3^C NMR *δ* (in ppm)	^1^ ^3^C / DEPT
1	45.8	47.1	CH_2_
2	68.0	67.9	CH
3	78.5	—	CH
4	42.3	42.2	Quaternary carbon
5	47.8	46.5	CH
6	17.8	17.5	CH_2_
7	32.5	32.6	CH_2_
8	38.9	38.9	Quaternary carbon
9	47.3	47.4	CH
10	37.6	37.8	Quaternary carbon
11	22.7	23.0	CH_2_
12	121.4	121.9	CH
13	144.1	144.3	Quaternary carbon
14	41.5	41.0	Quaternary carbon
15	29.3	27.6	CH_2_
16	23.7	23.5	CH_2_
17	46.3	45.9	Quaternary carbon
18	41.1	41.2	CH
19	45.7	46.1	CH_2_
20	30.9	30.9	Quaternary carbon
21	33.6	33.8	CH_2_
22	31.8	32.3	CH_2_
23	67.6	64.3	CH_2_
24	12.5	14.2	CH_3_
25	16.5	17.9	CH_3_
26	16.4	17.2	CH_3_
27	26.1	26.1	CH_3_
28	181.8	178.6	Quaternary carbon
29	32.7	33.3	CH_3_
30	23.1	23.76	CH_3_

The partial characterization of the ^1^ ^3^C peaks (Figure 10, Table 5) for SGA‐24 revealed different chemical shift regions: An aliphatic region from *δ* 14 to 47 ppm, and a region from *δ* 64 to 67.5 ppm corresponding to hydroxyl‐bearing methylene and methine groups. The solvent (DMSO‐d_6_) appeared between *δ* 38.9 and 40.4 ppm. Olefinic carbons and a carboxylic acid group were observed in the region of *δ* 121.9–178.6 ppm.

**FIGURE 11:**
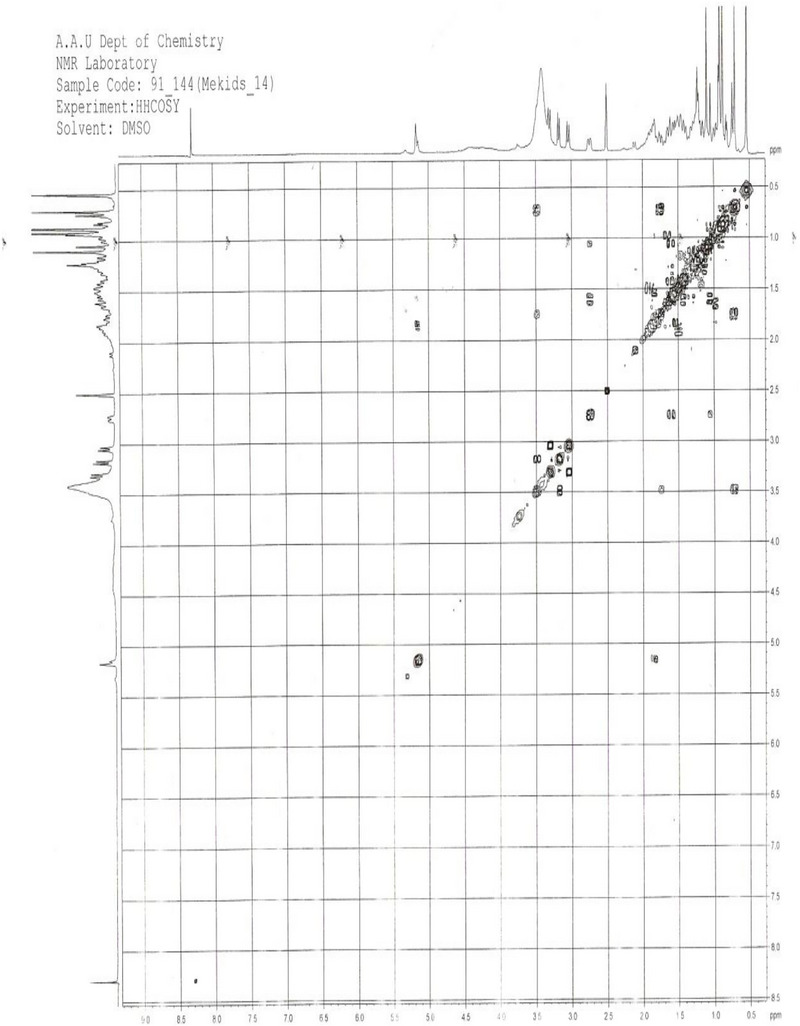
H‐H COSY spectrum of SGA‐24.

The DEPT‐135 spectrum (Figure 11, Table 5) of the partial structure of compound SGA‐24 provided detailed insights into its carbon framework, showing 21 carbon peaks out of the 29 carbons observed in the ^1^ ^3^C NMR spectrum. This analysis confirmed the presence of six aliphatic methyl singlets at *δ* 14.1 (C‐23), 17.2 (C‐25), 17.4 (C‐26), 23.75 (C‐30), 26.1 (C‐27), and 33.3 (C‐29) ppm, which fall within the expected range for methyl carbons in triterpenes.

Ten methylene groups were identified at *δ* 17.5 (C‐6), 23.0 (C‐11), 23.5 (C‐16), 27.6 (C‐15), 32.1 (C‐22), 32.9 (C‐7), 33.8 (C‐21), 46.1 (C‐19), 47.1 (C‐1), and 64.3 (C‐24) ppm. Notably, the signal at *δ* 64.5 (C‐24) indicated a methylene carbon attached to a hydroxyl unit, consistent with the downfield shift observed for carbons bearing oxygen substituents [40].

Five methine groups were found at *δ* 41.2 (C‐18), 46.5 (C‐5), 47.4 (C‐9), 67.9 (C‐2), and 121.9 (C‐12) ppm. The chemical shift at *δ* 67.5 (C‐2) specifically indicated a methine carbon attached to a hydroxyl unit, further supporting the presence of hydroxyl functionalities in the molecule. The olefinic methine carbon at *δ* 121.9 (C‐12) is also in good agreement with typical values for carbons in double bonds [35]

The difference in carbon count between the ^1^ ^3^C and DEPT‐135 spectra indicated the presence of eight quaternary carbon atoms in the partial structure of compound SGA‐24. These were observed at *δ* 30.9 (C‐20), 37.8 (C‐10), 38.9 (C‐8), 41.0 (C‐14), 42.5 (C‐4), 47.3 (C‐17), 144.3 (C‐13), and 178.6 (C‐28) ppm. Among these, the peak at *δ* 144.3 (C‐13) indicated the presence of a double bond, and *δ* 178.6 (C‐28) corresponded to a carboxylic acid group.

**C‐3 Position**: Based on Table 5, the chemical shift for the carbon at position 3 was not assigned in the partial structure of compound SGA‐24, likely due to unknown side chains attached at C‐3. However, 2D NMR data, particularly the HMBC spectrum, showed correlations of C‐3 with H‐1, H‐23, and H‐24 protons. In contrast, arjunolic acid from the literature features a hydroxyl group attached to a methine group at the third position, suggesting a structural difference for SGA‐24 at this position.

**FIGURE 12:**
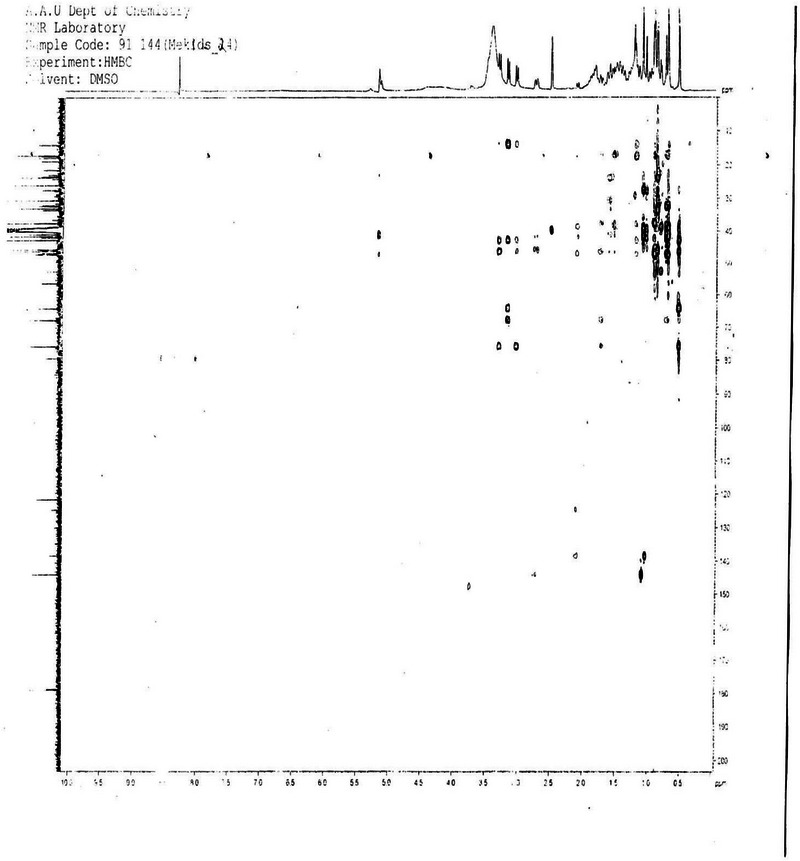
HMBC spectrum of SGA‐24.

**FIGURE 13:**
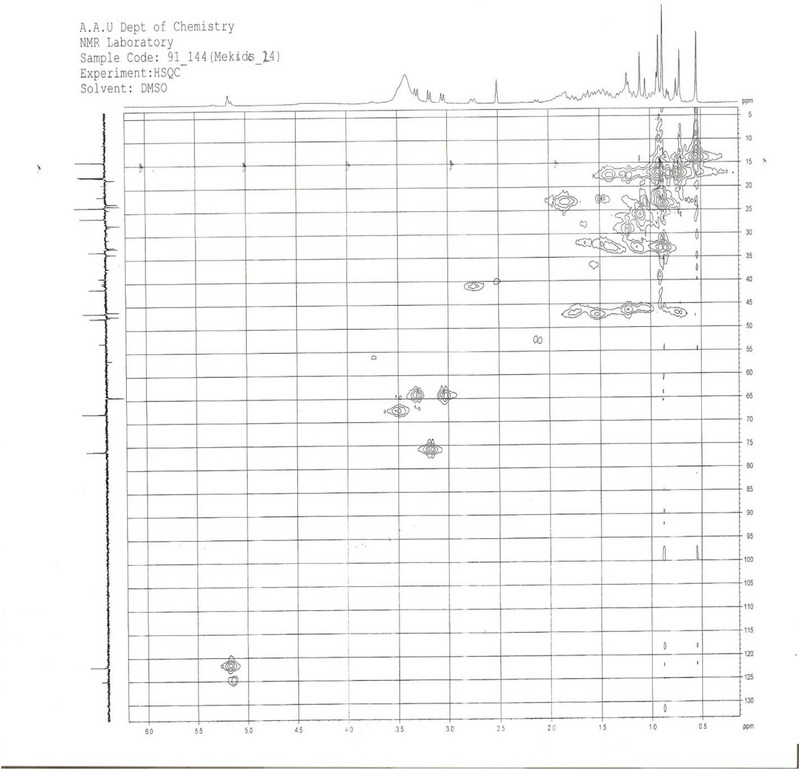
HSQC spectrum of SGA‐24. Based on the spectroscopic data the compound SGA‐24 is a terpenoid with the following partial structure.

**2D NMR (HMBC and HSQC) Analysis**: The unassigned chemical shift for C‐3 in compound SGA‐24, despite observed HMBC correlations with H‐1, H‐23, and H‐24, suggests a unique structural environment at this position that deviates from typical triterpene patterns (Figure 12). The precise assignment of carbon signals in complex natural products, particularly triterpenes with their intricate polycyclic skeletons and diverse side chains, remains a significant challenge in NMR spectroscopy. The HSQC spectrum of SGA‐24 directly correlated proton chemical shifts with their corresponding DEPT carbons (Figure 13). The protons at *δ* 3.29 and 3.01 ppm correlated with carbon at *δ* 64.5 ppm (C‐23), and the proton at *δ* 3.5 ppm correlated with carbon at *δ* 68.6 ppm (C‐2), confirming hydroxyl groups attached to methylene and methine units, respectively. The olefinic proton at *δ* 5.12 ppm correlated with carbon at *δ* 121.9 ppm (C‐12), indicating a ring olefinic double bond. Methyl proton signals at *δ* 0.54, 0.87, 0.86, 0.88, 0.89, and 1.16 ppm directly correlated with carbons at *δ* 14.1 (C‐24), 23.76 (C‐30), 33.3 (C‐29), 17.2 (C‐26), 17.9 (C‐25), and 26.1 (C‐27) ppm, respectively. While HMBC correlations are invaluable for establishing connectivity, the chemical shift of a carbon, especially at a key position like C‐3, can be profoundly influenced by the nature, stereochemistry, and even conformational dynamics of attached substituents [41, 42]. If SGA‐24 possesses an unusual side chain or an atypical functionalization at C‐3 that is not commonly found in known triterpenes, its ^1^ ^3^C chemical shift might fall outside expected ranges or overlap with other signals, making unambiguous assignment difficult without further advanced spectroscopic techniques. Recent advancements in NMR, such as the application of diffusion‐ordered spectroscopy (DOSY) to separate signals from mixtures, or computational approaches like density functional theory (DFT) calculations for predicting chemical shifts, are increasingly employed to resolve such ambiguities and confirm the structures of novel or highly modified natural products, especially when traditional 1D and 2D NMR data are insufficient for complete assignment [43]. Therefore, the unassigned C‐3 in SGA‐24, despite its clear HMBC correlations, strongly implies a structural novelty at this position, differentiating it from the well‐established hydroxylated methine at C‐3 in arjunolic acid [44]. The quaternary carbon C‐4 correlated with methylene protons H‐6, methyl protons H‐24, and methylene protons H‐23. Other significant correlations observed in Table 6 for HMBC are depicted in Figure 4.

**TABLE 6:** ^1^H, ^1^H‐^1^H COSY and HMBC spectra data of the partial characterization of compound SGA‐24.

Position	^1^ ^3^C/DEPT	^1^H NMR (ppm)	HMBC (C‐H)	^1^H‐^1^H COSY (H‐H)
1	CH_2_	1.75 (m), 0.74 (d)	25	H1‐H5
2	CH	3.5 (brS, 1H)	1	H2‐H1, H26
3	—	—	—	—
4	C	—	6, 23, 24	—
5	CH	0.71 (d, 1H)	23, 24	—
6	CH_2_	1.42 (m), 1.29 (dt)	5, 7	—
7	CH_2_	1.63 (m), 1.49 (m)	26	—
8	C	—	5, 26	—
9	CH	1.53 (m, 1H)	12, 25	H9‐H11, H19, H27
10	C	—	9, 11	—
11	CH_2_	1.50 (m), 1.49 (m)	—	—
12	CH	5.17 (t, 1H)	—	H12‐H1, H16
13	C	—	11, 18	—
14	C	—	12, 15, 27	—
15	CH_2_	1.61 (m), 0.92 (d)	27	—
16	CH_2_	1.81 (m), 0.93 (d)	15	H16‐H12
17	C	—	18, 19	—
18	CH	2.74 (d, 1H)	12, 22	H18‐H9, H15, H27
19	CH_2_	0.99 (d), 0.91 (d)	—	H19‐H21
20	C	—	18, 29	—
21	CH_2_	0.83 (d), 0.82 (d)	22, 30	—
22	CH_2_	1.65 (m), 1.49 (m)	—	—
23	CH_2_	3.01, 3.29 (d, 2H)	24	—
24	CH_3_	0.54 (s, 3H)	7, 23	H24‐H26
25	CH_3_	0.89 (s, 3H)	9	H25‐H24, H26
26	CH_3_	0.88 (s, 3H)	9, 11, 27	H26‐H24
27	CH_3_	1.16 (s, 3H)	—	H27‐H25, H29, H30
28	C	—	—	—
29	CH_3_	0.86 (s, 3H)	30	—
30	CH_3_	0.87 (s, 3H)	29	H30‐H24

#### 3.1.3 Comparison With Arjunolic Acid and Proposed Partial Structure

**FIGURE 14:**
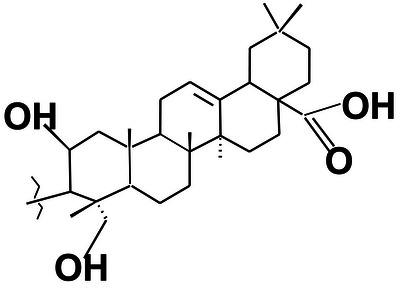
The partial structure of SGA‐24.

**FIGURE 15:**
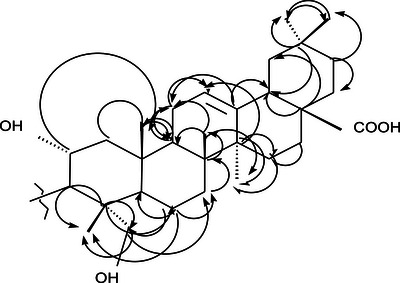
Major HMBC correlation in the partial structure of SGA‐24.

**FIGURE 16:**
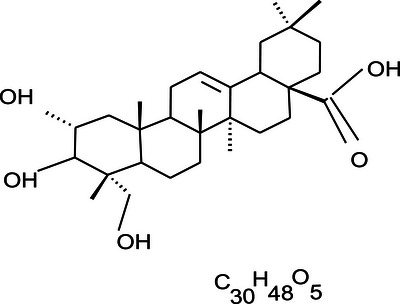
Arjunolic acid.

Based on HSQC and HMBC correlations, the characteristic chemical shift for the ring olefinic proton at *δ* 5.12 ppm was assigned as H‐12, as it correlated with carbons C‐9 and C‐16. The carbon–carbon double bond C‐13 at *δ* 144.3 ppm correlated with protons H‐18 and H‐16. Therefore, the olefinic double bond was placed between C‐12 (*δ* 121.9 ppm) and C‐13 (*δ* 144.3 ppm), suggesting the presence of an oleanane triterpene with a Δ12‐ene skeleton. This finding is further supported by the close similarity in chemical shifts between the partial structure of SGA‐24 (Figure 14) and arjunolic acid (Figure 16), a known oleanane‐type triterpene [45]. The major HMBC correlations observed for SGA‐24 are illustrated in Figure 15. The structural similarities observed in the NMR data, particularly the oleanane skeleton and the presence of a carboxylic acid group, are consistent with previous reports on triterpenoids isolated from *Syzygium* species and other medicinal plants. While the full structure of SGA‐24 requires further elucidation due to unassigned carbons, the partial structure strongly suggests its classification as a terpenoid.

## 4 Conclusion

This study successfully investigated the phytochemical constituents of the root extract of *S. guineense* from South Ethiopia, a region where detailed phytochemical studies on this plant part have been limited. Through systematic solvent extraction, preliminary phytochemical screening confirmed the presence of various secondary metabolites, including alkaloids, flavonoids, terpenoids, polyphenols, saponins, tannins, glycosides, and steroids, supporting the plant's traditional medicinal applications. Subsequent column chromatography and TLC analysis led to the isolation and partial characterization of two pure compounds: SGB‐3 from the methanol extract and SGA‐24 from the chloroform extract. Spectroscopic data (IR, ^1^H NMR, ^1^ ^3^C NMR, and DEPT) strongly indicated that SGB‐3 is a diterpene lactone. For SGA‐24, comprehensive 1D and 2D NMR analyses (HMBC, HSQC) revealed its partial structure as an oleanane Δ^1^ ^2^‐ene triterpenoid, exhibiting significant similarities to the known compound arjunolic acid. These findings provide crucial scientific validation for the traditional uses of *S. guineense* roots in the Wolaita Zone and lay a foundational chemical profile for this specific plant material, highlighting its potential as a source of bioactive natural products.

## Conflicts of Interest

The author declares no conflicts of interest.

## Data Availability

The data that support the findings of this study are available from the corresponding author upon reasonable request.
